# Balanced Energy Protein Supplementation in Pregnancy: Adherence and Acceptability among Pregnant Women in Rural Ethiopia

**DOI:** 10.1016/j.cdnut.2024.103796

**Published:** 2024-06-13

**Authors:** Michelle Eglovitch, Firehiwot Workneh Abate, Tigest Shifraw, Fisseha Shiferie, Hanna Amanuel, Amare Worku Tadesse, Alemayehu Worku, Sheila Isanaka, Yemane Berhane, Anne CC Lee

**Affiliations:** 1 Brigham and Women’s Hospital, Boston, MA, United States; 2 Virginia Commonwealth University, Richmond, VA, United States; 3 Addis Continental Institute of Public Health, Addis Ababa, Ethiopia; 4 Harvard Medical School, Boston, MA, United States; 5 London School of Hygiene and Tropical Medicine, London, United Kingdom; 6 Harvard T.H. Chan School of Public Health, Boston, MA, United States

**Keywords:** acceptability, adherence, balanced energy protein supplement, corn–soy blend, pregnancy, Ethiopia, fortified blended foods

## Abstract

**Background:**

Balanced energy protein (BEP) supplementation in pregnant women in low-and middle-income countries may reduce the risk of stillbirth and low birth weight.

**Objectives:**

The objective of this study was to assess the adherence to and acceptability of a corn–soy blend (CSB) BEP product among pregnant women in rural Ethiopia.

**Methods:**

This formative study was conducted from October to November 2018 among pregnant women in the rural Amhara region of Ethiopia prior to initiation of a clinical effectiveness study (ISRCTN: 15116516). We assessed adherence and acceptability of a micronutrient-fortified CSB BEP supplement among 40 pregnant women during a 4-wk utilization period. Acceptability was assessed using a 7-point Likert-style scale about the hedonic characteristics of the BEP product at 2 wk and 4 wk. Adherence was assessed by weekly monitoring and empty sachet counts for BEP consumption over 4 wk.

**Results:**

Adherence to the BEP was, on average, 89% over the month-long pilot. The BEP product was rated favorably (mean Likert score >6 of 7) for the following domains: color, taste, odor, and likeability at mid and endpoints. Women found the product convenient to eat [mean (standard deviation [SD] = 5.9 (1.0))] and filling (mean (SD) = 6.1 (1.5) out of 7). Scores on acceptability and perception of the product remained stable throughout the duration of use. A majority of women (63%) reported consuming the BEP as a snack to supplement meals and splitting the serving over 2 or more sessions (68%). A quarter of respondents reported sharing the supplement with family members.

**Conclusions:**

Adherence and acceptability of the CSB BEP product were high among this population in rural Amhara, Ethiopia. This formative data was important to select the final product and shape the counseling and delivery of BEP in the parent study.

## Introduction

Being born with low birth weight (LBW) (<2500 g) due to preterm birth or fetal growth restriction is a significant risk factor for infant mortality and contributes to 60–80% of neonatal deaths worldwide [[1], [2], [3]]. Maternal underweight status (defined as BMI [in kg/m^2^] <18.5) increases the risk of preterm birth (<37 weeks gestational age), as well as being born LBW and small for gestational age (<10% birthweight for gestational age and sex) [4]. Babies born LBW, preterm, and small for gestational age have an increased risk of childhood stunting, which results in an intergenerational cycle of sub-optimal growth and development [5]. Maternal undernutrition is prevalent in the rural region of Amhara, Ethiopia, where an estimated 23% of women of reproductive age have a BMI <18.5 [6]. Further, maternal short stature as a result of chronic maternal undernutrition is a risk factor for adverse birth outcomes, which is consistent with the hypothesis that lack of quality nutrition can contribute to intergenerational cycles of poor growth and development [7].

Prenatal balanced energy protein (BEP) supplements (nutrition supplements providing <25% of total energy from protein) may positively impact birth outcomes. In a Cochrane meta-analysis, BEP supplementation increased mean birthweight by 41 g and reduced the risk of stillbirth [8]. The WHO antenatal care guidelines recommend the provision of BEP supplements in populations where the prevalence of BMI <18.5 is >20% [9]. The Ethiopian Ministry of Health (MOH) recommends that pregnant women with acute malnutrition, defined as middle upper arm circumference (MUAC) <23 cm, be provided with nutritional supplementation during pregnancy [10]. In Ethiopia, the management of acute malnutrition includes a targeted supplementary feeding program available in certain famine and drought-prone areas, wherein a daily ration of corn–soy blend (CSB) is provided. However, in practice, implementation of this recommendation is limited due to resource constraints.

To increase the reach and coverage of BEP supplementation interventions, formative research is needed to understand the factors that may affect acceptability, perception, and adherence to specific supplements in different contexts. This article presents results from a formative study conducted prior to the initiation of the “Enhancing Nutrition and Antenatal Infection Treatment” study in the rural Amhara region of Ethiopia (ISRCTN: 15116516). The objective of this formative study was to assess the acceptability of, and adherence to, a proposed BEP supplement among pregnant women prior to initiation of a pragmatic clinical effectiveness study.

## Methods

### Study site

This acceptability study was the first part of a pragmatic clinical effectiveness study to evaluate the effect of optimizing maternal nutrition and antenatal infection management on maternal and birth outcomes in Ethiopia [11]. The study site was in the West Gojjam and South Gondor districts of the Amhara region of Ethiopia, which has low-resourced health systems and poor health indicators. Per 2016 Ethiopian Demographic Health Services data, Amhara had the country’s highest rates of neonatal mortality (47/1000 live births) and LBW (22.2%) and a high maternal mortality rate (412/100,000 live births) [6].

### Study participants

Participants were recruited from 4 rural health centers in West Gojjam and South Gondor, Amhara, from October to November 2018. Research staff recruited pregnant women identified as being undernourished by a MUAC of <23 cm who visited 1 of 4 health facilities for prenatal care. Women were invited to participate until the sample size was met (*n* = 40). Inclusion criteria included being pregnant in the second trimester as identified through antenatal care records, having a MUAC <23 cm, living in health center catchment areas, and intention to stay in study areas for ≥1 mo. A sample size of 40 was used as it was considered sufficient to reach saturation in the open-ended responses.

### Study design and procedures

This formative research was a prospective pilot study that collected quantitative data on BEP supplement acceptability, perceptions, and adherence among participants.

#### BEP product overview

Multiple factors were considered in selecting the BEP supplement included in the parent study, including community and stakeholder preferences and religious beliefs. The rural Ethiopian community in the Amhara region is primarily Orthodox Christian, which stipulates a vegan diet, therefore eliminating the use of certain dairy-containing products during fasting [12]. Prior to the pilot study, several different BEP supplements were tested by local community members and Ethiopian MOH stakeholders, including a vegan lipid-based ready-to-use nutrition supplement [lipid-based nutritional supplement (LNS), Plumpy Mum; Nutriset] with several different flavors (standard savory, and tomato-onion) and the current vegan, micronutrient-fortified CSB product, a blended flour made of corn and soybeans and fortified with minerals and vitamins (Super Cereal; Faffa Food Products) [13].

After several tasting sessions, local community members and stakeholders from the MOH expressed a preference for the familiar, well-liked, and locally produced CSB. Community stakeholders did not prefer the taste or texture of the Plumpy Mum LNS product, and ministry stakeholders had a strong inclination for the CSB over the foreign-produced LNS, considering future production and scale-up capacity. Accordingly, the fortified CSB for this study was produced locally by Faffa Foods SC, located in Addis Ababa, and packaged into daily 75 g servings for this pilot study (nutritional contents outlined in Supplemental Table 1). The 75 g portion was chosen due to its alignment with recommendations from an expert convening on specifications of food supplements for pregnant and lactating women [14].

#### BEP distribution

BEP was distributed at the 4 study health centers at enrollment and at or near the study health centers for 3 subsequent weeks. At each weekly distribution visit, women received 7 sachets of BEP. Subsequent distributions occurred either at home or at the study health center based on the participant’s preference. Study staff counseled the women to consume the BEP daily as a supplement, not as a replacement for usual meals. They were provided recipes for the preparation of the supplement and instructed to mix the flour with warm water over a stove to create a porridge-like product. They were instructed that after preparation, they could add oil or other additives per dietary preference.

Women were informed that the BEP could be eaten in multiple portions throughout the day but needed to be completed by the end of the day. In situations where the mothers could not consume the whole portion in one sitting, they were counseled to cover the prepared mix until consumption within the same day and discard leftovers. Participants were counseled that the BEP supplement was a treatment for the mother, that it would help provide specific nutrition for the growing infant, and that the product should not be shared with members of their household or with their neighbors. The 75-g sachets were provided for free to the mothers.

#### BEP adherence monitoring

Research staff conducted weekly visits to monitor BEP adherence. Study staff called participants to remind them to come to the health center for follow-up visits, and those who were unable to return to the health center were visited at home. Adherence was assessed using sachet count; participants were asked to return used/empty BEP sachets at each distribution visit, and study staff counted the empty sachets from the last distribution.

#### BEP acceptability assessment

Study staff administered questionnaires to assess the acceptability of this supplement after 2 and 4 wk of utilization based on its hedonic properties using a 7-point Likert scale (0 = strongly disagree/dislike very much to 7 = strongly agree/like very much). Questionnaire data was collected using the Open Data Kit software (ODK, Inc.) Acceptability domains included color, taste, texture, odor, portion size, ease of use, and overall likeability. Perceived child-likeability was used to assess the extent to which a participant believed her child would like the product and used to provide insight into the potential for intrahousehold sharing in the main trial. Participants also rated their perception of the product use in terms of being convenient to eat, the product being medicine or food or both, and feeling full after eating a full serving. Study staff also elicited responses to open-ended questions about how perceptions of the product changed over time and why, as well as probed for suggestions to improve the product for future use. Responses were recorded by study staff on secure tablets.

### Data analysis

We analyzed the 7-point Likert scale used for quantification of product acceptability and perceptions as a continuous variable [15]. The means (SDs) were calculated for the characteristics of acceptability and perception of product use. We also calculated the percentage of women who scored each question as favorable, defined as a score ≥5. Adherence was calculated as the proportion of sachets consumed by empty returned sachet count. The adherence proportion was defined as the number of sachets consumed (used sachets returned) divided by the total number of sachets distributed. Women who were lost to follow-up (LTFU) were included in the analysis as having 0 sachets consumed for the week(s) they were LTFU. A paired t-test was used to assess differences in acceptability and perception domains at 2 and 4 wk. The *z*-score tests were used to detect differences in adherence proportions over the 4-wk time period. Statistical analysis for quantitative data was done using Stata version 17 (StataCorp LLC, 2021). Open-ended questionnaire data were translated by study staff from Amharic into English using Microsoft Word. The responses were then analyzed in English using inductive content analysis, meaning that themes arose naturally from the data, which were identified by the study authors [16].

### Ethical statement

The study was approved by the ethics committees of the Addis Continental Institute of Public Health in Addis Ababa, Ethiopia, and Partners HealthCare/Mass General Brigham in Boston, Massachusetts, United States. Additional permission was obtained from the Amhara Regional Health Bureau, zonal health departments, and the study health centers. The study was explained to potential study participants, and participants willing to take part completed the consent form with a signature or thumbprint.

## Results

### Participant characteristics

Participants were, on average, 26.3 + 3.9 y old. The median (IQR) gestational age of pregnancy of the participants at enrollment was 24 wk [17,18]. The average MUAC was 21.7 cm at enrollment (SD 1.1; range 19.5–23 cm).

### BEP adherence

Forty women were enrolled in the adherence study, and we completed BEP adherence home visits among 37 (92.5%), 37 (92.5%), and 33 (82.5%) women at 2, 3, and 4 wk, respectively. Adherence by week is shown in Table 1. The average number of sachets consumed over the prior week was high throughout the follow-up. Women consumed, on average, 80–90% of sachets across the 4-wk period.

**TABLE 1:** Adherence to balanced energy protein supplement (*n* = 40 women)

	Average number of sachets consumed (out of a total of 7)	Average adherence (% of sachets consumed)
Week 1 (*n* = 40)	6.4 (1.4)	91.1
Week 2 (*n* = 37)	6.2 (2.1)	88.9
Week 3 (*n* = 37)	6.5 (2.1)	92.9
Week 4 (*n* = 33)	5.7 (2.7)	81.4
Overall	6.2 (2.1)	88.6

Overall, adherence was high among the study participants who completed the follow-up (*n* = 33). Among those who were followed up the entire period *(n* = 33), the overall average adherence was 91% in week 1, 95% in week 2, 100% in week 3, and 99% in week 4. However, in order to conservatively report adherence by assuming those LTFU did not consume any BEP across all 40 participants, the overall adherence percentage was 91% in week 1, 89% in week 2, 93% in week 3, and 81% in week 4 (Table 1). Adherence did not significantly change from week 1 to week 2 (*P* = 0.764), week 2 to week 3 (*P* = 0.529), or week 3 to week 4 (*P* = 0.110).

### BEP consumption patterns

Nearly half of the women (15/35; 42.9%) reported consuming the sachet in 1 sitting, whereas 13/35 women (40.0%) reported splitting it into 2 portions, and 6 (17.1%) reported splitting it into 3 or more portions throughout the day. Although more than one-third of women (*n* = 13/35, 37.1%) reported using the sachet as part of a larger meal, typically breakfast and supper, the majority of women (*n* = 22; 62.9%) consumed the sachet as a snack during the day.

### BEP acceptability and product perception

Participant responses to the scale responses on the acceptability and perceptions of the BEP are shown in Table 2. Generally, the BEP was rated to have high acceptability (mean scores for sensory items ranging 5–6 on a Likert scale out of a maximum of 7). The highest mean scores were for color and odor at 2 and 4 wk. The product acceptability remained stable for most domains across both timepoints, with significant improvements (*P* < 0.05) in scores for color and ease of use/consumption.

**TABLE 2:** Acceptability of balanced energy protein at week 2 and week 4 (on a 7-item Likert scale)^1^

Acceptability				
	Week 2 (*n* = 37)		Week 4 (*n* = 33)	
	Mean (SD)	*n* (%) reported like^2^	Mean (SD)	*n* (%) reported like^2^
Color	6.3 (1.2)	97.5	6.5 (0.9)	97.0
Taste	5.9 (1.3)	89.7	5.9 (1.2)	84.8
Texture/consistency	5.3 (1.3)	82.1	5.1 (1.3)	78.8
Odor	6.1 (1.3)	87.2	6.4 (0.9)	91.0
Portion size	5.8 (1.5)	82.1	6.1 (1.0)	93.9
Ease of use/consumption	5.5 (1.2)	84.2	5.7 (1.2)	84.4
Overall likeability	5.2 (1.3)	74.4	5.3 (1.1)	81.8
Perceived child-likeability	5.4 (1.2)	82.1	5.2 (1.2)	72.7

Abbreviation: SD, standard deviation.

1 1 = dislike very much, 7 = like very much.

2 Likert score ≥5.

We also assessed study participants’ perceptions of the product (Table 3). The BEP product was generally found to be filling and convenient to eat. Participants also ranked the BEP product highly for being willing to use it daily during pregnancy if provided at no cost. These perceptions remained stable throughout the month of follow-up and did not significantly change over the course of the month.

**TABLE 3:** Perception of balanced energy protein at week 2 and week 4 (on a 7-item Likert scale)^1^

Perception	Week 2 (*n* = 37)		Week 4 (*n* = 33)	
	Mean (SD)	% reported agree^2^	Mean (SD)	% reported agree
Product is convenient to eat	5.5 (1.4)	82.1	5.9 (1.0)	90.1
Product is medicine	4.2 (2.2)	56.4	4.1 (2.3)	54.5
Feel full after full portion	6.2 (1.4)	87.2	6.1 (1.5)	81.8
Would share with others	3.7 (1.2)	48.7	4.2 (2.1)	51.5
Would use daily if provided freely	6.0 (1.5)	87.2	6.3 (1.0)	90.9

Abbreviation: SD, standard deviation.

1 1 = strongly disagree, 7 = strongly agree.

2 Likert score ≥5.

### BEP sharing

More than a quarter (26%) of respondents reported sharing the supplement with family members. In addition, 17/35 (48.6%) respondents indicated that other members of the household expected them to share the product.

### BEP preferences regarding future use

Regarding future use, 62.9% (22/35) reported that if they were to use the product during a future pregnancy, they would use it “always.” In addition, 62.9% (22/35) of women indicated they would prefer once or twice monthly deliveries of a future supplement instead of weekly, and 74.2% (26/35) of the women indicated that they would like to receive the supplement at the health center or nearby health post.

### Open-ended questionnaire feedback

#### Theme 1: perception of the product changed over time

Some women reported evolving preferences for the BEP supplement:

“At first, I was fed up with eating the supplement, but over time I liked it.” -ID SALA02

“At first, I didn’t like it because it was new for me, but now, I like it.” -ID SALA06

Some women also mentioned that they initially did not consume the entire sachet daily, but after time and use, they increased their consumption to consume the entire packet:

“At first, I didn’t finish the whole portion, but now, I am eating the whole package.” -ID SALA05

“At first, I didn’t finish the whole sachet, but now I like it.” -ID SALA07

They also mentioned that counseling was an important part of acclimating:

“Over time, I understood its use and consumed more.” -ID LKYIF06

#### Theme 2: suggestions for improvement of the product

Women provided specific suggestions to improve the product so that it would be more acceptable in the future.

“The texture of the product is coarse. It is better if it can be modified to a finer powder.” -ID LKYIF08

“The odor needs to be modified, and it will be better if the packaging can be modified as well as it can be torn up easily” -ID SALA06

#### Theme 3: BEP sharing

Finally, 20/35 women (57.1%) indicated that if they ate the BEP in the future, they would share it with family members, particularly children and husbands. When asked why they would share the product in the future, women mentioned the stigma around eating alone in their communities.

*“Eating alone is taboo in our community.”* -*ID DEANB06*

Women also indicated that they would share the BEP product due to considerations around their children.

*“I would share the product because I don’t want my children to get malnourished.”* -*ID SALA03*

“I want to give to my children because they think it’s a different thing and it has good taste.” -ID LKYIF10

Overall, open-ended responses were consistent with the quantitative survey results. Although a few women reported that initially, they did not like the product or think that they would use it, over time, it became easier to use, and their preference for the product improved. A few (*n* = 6) women mentioned that the BEP odor made them feel nauseous; however, one woman noted that the nausea was related to general pregnancy.

## Discussion

In this formative study, we aimed to assess adherence to and acceptability of a BEP supplement among undernourished pregnant women in rural Amhara, Ethiopia. During this formative study, BEP adherence rates were high throughout the full 4-wk period (80–90%). Participants reported positive responses to hedonic characteristics, including color, taste, and overall likeability over the duration of the study. Most women in the study sample indicated that they used the BEP to supplement their meals. ∼1 quarter of participants shared the supplement with family members.

The CSB BEP supplement tested in our study population had high acceptability in this context. The BEP supplement scored favorably with respect to its sensory properties (taste, smell, and color). In Ethiopia, the CSB BEP product is a common food supplement delivered by the Ethiopian MOH and non-governmental organizations (NGOs), and the local community is accustomed to the taste and preparation of the product. Although the CSB was not ready-to-use, participants were familiar with the product and found it easy to prepare, scoring highly on the usability domain and convenient to eat. In addition, unlike other ready-to-use food supplements that contain dairy or peanut ingredients, the CSB BEP product is vegan, which aligns with Ethiopian Orthodox Christian practices requiring a vegan diet on most days of the year [12,19]. Thus, the CSB BEP product was acceptable in this population due to the favorable hedonic properties, local product familiarity, and alignment with local religious requirements.

In other low- and middle-income settings, the acceptability of CSB BEP supplements has been less favorable. Pregnant women in rural Cambodia disliked the smell produced during the boiling process, and the majority reported that the taste was bland and flavorless, which may have been particularly exacerbated by periods of morning sickness [20]. In rural Haiti, Beckett et al. [21] found that CSB BEP was less acceptable in comparison to other BEP products, such as ready-to-use supplementary food. Finally, in Burkina Faso, a preference for lipid-based nutrient supplements was also reported over CSB BEP [17]. These findings regarding the acceptability of CSB BEP emphasize the importance of local context and customs and the necessity of formative research to understand population-specific preferences prior to implementing nutrition interventions.

Several BEP efficacy trials participating in the Maternal BEP Harmonization initiative have used different BEP supplements and reported high levels of adherence [22]. For example, a home BEP supplement trial of a lipid-based peanut paste and vanilla biscuit that occurred over an 8-wk period of weekly product distribution in Burkina Faso found high adherence to both BEP supplements (99.8% and 99.6%, respectively) [23]. Similarly, a trial done in rural Nepal over an 8-wk period found adherence medians of 91.1% in the lipid-based peanut paste group and 96.4% in the vanilla biscuit group [19]. Of note, the current pilot and these reported studies were conducted with high-intensity monitoring and may not reflect adherence in a pragmatic real-world setting.

Sharing of the daily serving was an important consideration to inform the parent trial design, given that sharing the product may have reduced intake for women. Prior to distribution, participants were counseled not to share and to consume the whole daily serving as a supplement to usual meals. Despite this counseling, around a fourth of women reported sharing at some point during the 4 wk period. These findings align with previous research, which has demonstrated that CSB is more likely to be shared compared to other BEP supplements [24]. Wang et al. [24] posited that CSB may be more readily shared as it is porridge-like, which can be acceptable to children as porridge plays an important role in childhood feeding.

The likelihood of sharing BEP is important to consider, although the risk must be balanced with other considerations of food supplements in this population, particularly given the vegan diet that this population practices. Based on this formative work, for the main study, we developed counseling regarding the importance of the BEP for pregnant women, like a medicine for the growing infant, and also provided an additional supplement to account for anticipated family sharing [11]. This highlights the importance of counseling alongside product delivery to encourage proper utilization of the supplement.

Food displacement is a concern with BEP supplementation [25,26]. However, a fellow trial in the Maternal BEP Harmonization initiative that provided a similar amount of BEP (72-g) found that such supplements increased nutrient intake without displacing food intake [27]. We conducted extensive counseling regarding the use of the BEP as a supplement to be given in addition to regular meals, with counseling on the consumption and storage of unused products between meals. Thus, this formative research emphasized the role of the approach to counseling to reiterate key messaging and the importance of engaging health care providers in nutrition interventions.

### Limitations

As most measures in this study were self-reported, it is possible that participants may have expressed answers they perceived to be desired by the research staff, which may have resulted in the underreporting of negative responses. In addition, study attrition may have been affected by political and civil unrest in the Amhara region where the study was conducted, which may have impacted the willingness of women to fully follow-up with the study.

Another limitation of our study includes the shorter duration of observed adherence (4 wk). The 4-wk observation period was attainable and done in harmonization with 2 other partner BEP pregnancy trials with similar study procedures [19,22,23]. Although our study evaluated adherence over a shorter amount of time, a 4-wk period of daily consumption may be sufficient to examine acceptability as other studies have found that decreased motivation may appear after 3 wk as a function of the number of times a food is consumed [23,28,18]. Finally, we did not collect information about the proportion of the daily supplement that was consumed and if there was a leftover or remaining dose.

In conclusion, overall, the BEP supplement demonstrated high acceptability among pregnant women in rural Amhara, Ethiopia. The supplement was identified by the local community as a familiar product that was compatible with local religious beliefs and requirements during fasting seasons and had high favorability with respect to the taste, color, and odor of the product. During the acceptability study, we observed high levels of adherence at an average of 89% over 4 wk. The high acceptability helped finalize the selection of this CSB BEP product used in the parent study, and the formative findings shaped the delivery and counseling related to BEP distribution, including counseling to encourage proper use of the product (i.e., as a food supplement compared with meal replacement) and to address potential sharing practices [11].

## Author contributions

The authors’ responsibilities were as follows – AWT, AW, SI, YB, ACCL: conceived the study design; FWA, TS, HA, FS: were involved with the training of the study team; FWA, TS, FS: led the field team and supervision of the data collection; ME: analyzed the data and prepared the first draft of the manuscript; All authors contributed toward the review and finalization to the study design, study tools, and revisions of the manuscript; and all authors: read and approved the final manuscript.

## Conflict of interest

The authors report no conflicts of interest.

## Funding

This study was supported by The Bill and Melinda Gates Foundation (Principal Investigator: ACCL, OPP1184363).

## Data availability

Data described in the manuscript, code book, and analytic code will be available upon request, pending application and approval.
