# Supplementary material for: Balanced Energy Protein Supplementation in Pregnancy: Adherence and Acceptability among Pregnant Women in Rural Ethiopia
Source: Curr Dev Nutr. 2024 Jun 13;8(7):103796. doi: 10.1016/j.cdnut.2024.103796 (PMC11269774; doi:10.1016/j.cdnut.2024.103796)
Supplement: Multimedia component 1 [file mmc1.docx]

**Balanced Energy Protein Supplementation in Pregnancy: Adherence and Acceptability among Pregnant Women in Rural Ethiopia**

Authors: Michelle Eglovitch, MPH, MS^1^; Firehiwot Workneh Abate, MPH^2^; Tigest Shifraw, MPH^2^; Fisseha Shiferie, MPH^2^; Hanna Amanuel, MS^3^; Amare Worku Tadesse, MD, PhD^2,4^; Alemayehu Worku, MSc, PhD^2^; Sheila Isanaka, ScD^5^; Yemane Berhane, MD PhD^2^; Anne CC Lee, MD, MPH^1,3^

**6 APPENDIX**

Supplementary Table 1: Nutritional Composition of Study CSB BEP per 75g sachet

| Energy | 285 kcal |
| --- | --- |
| Protein | 10.5g |
| Fat | 5% |
| Vitamin A | 778.5 mcg |
| Vitamin D | 8.3 µg |
| Vitamin E | 6.3 mg |
| Vitamin K | 22.5 µg |
| Thiamin, B1 | 0.2 mg |
| Riboflavin, B2 | 1.1 mg |
| Niacin, B3 | 6 mg |
| Vitamin, B6 | 0.8 mg |
| Folic Acid, B9 | 82.5 µg |
| Vitamin B12 | 1.5 µg |
| Vitamin C | 67.5 mg |
| Iron | 3 mg |
| Zinc | 3.8 mg |
| Iodine | 30 µg |
| Calcium | 271.5 mg |
| Phosphorus | 210 mg |
